# Surgical Application of the Suboccipital Subtonsillar Approach to Reach the Inferior Half of Medulla Oblongata Tumors in Adult Patients

**DOI:** 10.3389/fsurg.2015.00072

**Published:** 2016-01-13

**Authors:** Alejandra T. Rabadán, Alvaro Campero, Diego Hernández

**Affiliations:** ^1^Division of Neurosurgery, Institute of Medical Research A. Lanari, University of Buenos Aires, Buenos Aires, Argentina; ^2^Department of Neurosurgery, Hospital Padilla, Tucumán, Argentina

**Keywords:** subtonsillar approach, medulla oblongata, tumors, brainstem, surgery

## Abstract

Medulla oblongata (MO) tumors are uncommon in adults. Controversies about their treatment arise regarding the need for histological diagnosis in this eloquent area of the brain, weighing benefits of a reliable diagnosis, and the potential disadvantages of invasive procedures. As a broader variety of pathological findings could be found in this localization, the accurate histopathological definition could not only allow an adequate therapy but also can prevent the disastrous consequences of empiric treatments. There are few publications about their surgical management and all belongs to small retrospective cohorts. In this scenario, we are reporting two patients with exophytic or focal lesions in the inferior half of the medulla, who underwent surgery by suboccipital midline subtonsillar approach. This approach was not specifically described to reach MO before, and we found that the lesions produced a mild elevation of the tonsils providing a wide surgical view from the medulla to the foramen of Luchska laterally, and up to the middle cerebellar peduncle, offering a wide and safe access.

## Introduction

Brainstem tumors are infrequent in adult patients. Median age of presentation is the fourth decade of life, and overall survival is about 54–64 months. They represent <2% of all brain tumors in epidemiological data (1). Those located exclusively in the medulla oblongata (MO) are still more sporadic, so that few reports of purely medulla tumors ranged from 0 to 21% among the brainstem tumors reported series (1–8) (Table 1).

**Table 1:** **Relation between number of tumors of the brainstem and medulla oblongata in adults**.

Reference	No. of BS tumors	No. of MO tumors
Grigsby et al. (2)	32	3
Shieve et al. (5)	19	0
Guiney et al. (4)	21	0
Landolfi et al. (3)	19	4
Selvapandian et al. (6)	30	0
Guillamo et al. (1)	48	12
Salmaggi et al. (8)	34	6

There are isolated reports of small surgical experiences dealing with direct surgery in this particular CNS region. Although most of them reported satisfactory results, others described that postoperative neurological deterioration was common and suggested that surgery is of questionable benefit (9–18).

Regarding the type of mass lesions that can be found in adults, a diversity of histopathological findings has been reported, such as high-grade and low-grade astrocytomas, anaplastic and low-grade oligodendrogliomas, ependymomas, gangliogliomas, DNT’s, metastases, granulomas, lymphomas, infection, inflammatory diseases, and pseudotumoral lesions as tumefactive demyelinating diseases (1, 19, 20). These oncological and non-oncological entities require specific treatment and obviously have a very different prognosis.

Therefore, controversies arise regarding the need for histological diagnosis in this eloquent area of the brain, weighing benefits of a reliable diagnosis and the potential disadvantages of invasive procedures. In this scenario, recommendation of the surgical management in adult patients cannot be defined with an appropriate level of evidence. Meanwhile, the contribution of our two cases experience dealing with uncommon tumors of the MO can provide more information for the surgical decision making process.

## Patients and Methods

### Report of Cases

#### Case 1

A 41-year-old woman had a 6-month history of headache, vertigo, unsteadiness, and persistent cough. At admission, she had 90 points Karnofsky performance status (KPS). The patient did not have a relevant medical history and her clinical work up was normal. Funduscopy was normal. T1-weighted MRI scans showed an exophytic hypointense and homogenous mass in the left MO without gadolinium enhancement. Complete MRI scans of the entire neuroaxis were unremarkable. The suspected preoperative diagnosis was low-grade glioma or ependymoma, according to the more common lesions with its radiographic features in this CNS localization. The intraoperative biopsy demonstrated a tumor composed of cells like ependymoma and astrocytes. This finding supported the complete excision of the lesion. The definitive diagnosis was subependymoma.

**Figure 1:**
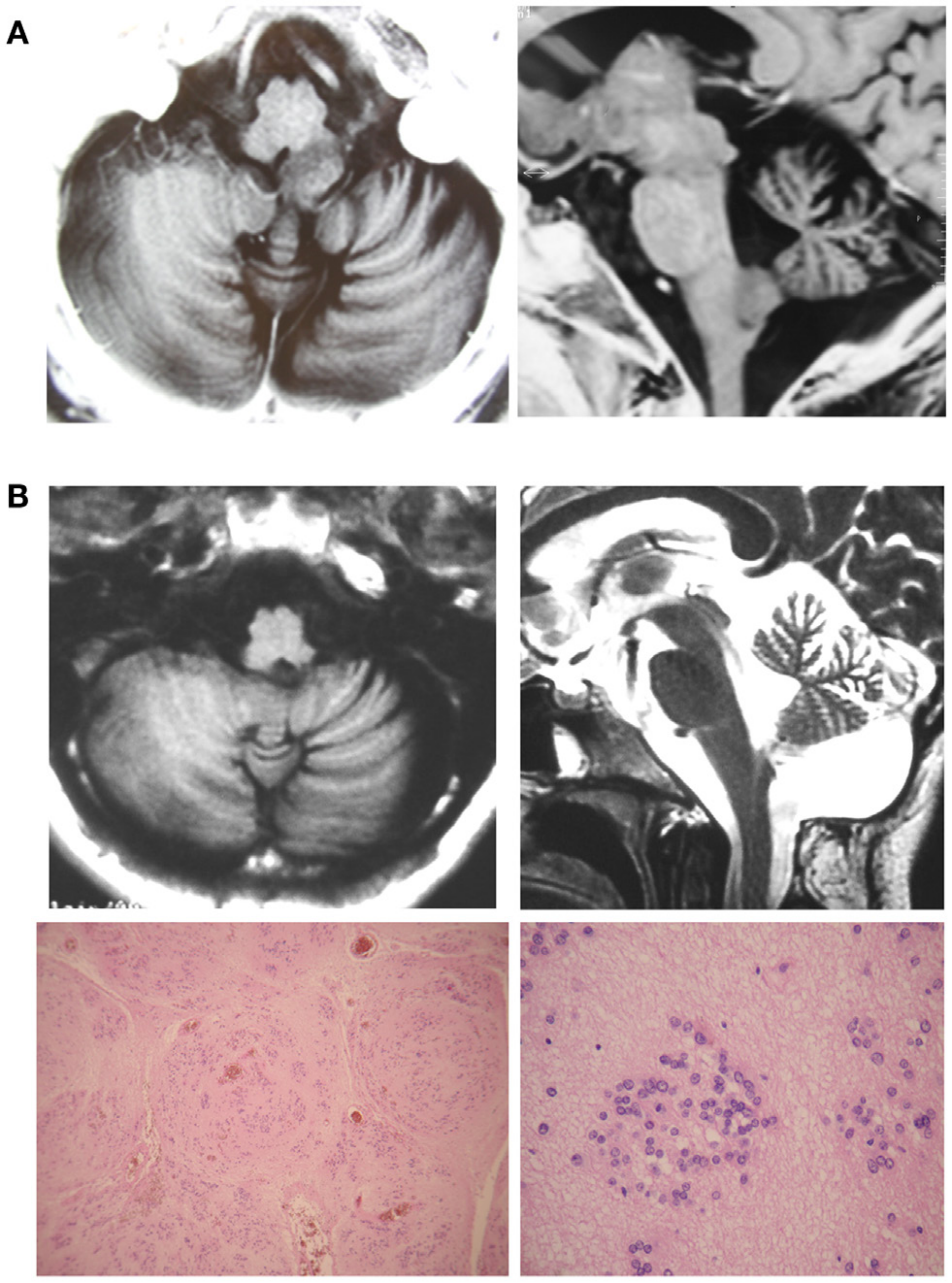
**(A)** Case 1. Preoperative T1-weighted brain MRI scan showed a hypointense and homogenous mass within the left MO without Gadolinium enhancement. **(B)** The postoperative T1-weighted brain MRI scan showed complete resection of the tumor.

Postoperatively, the patient presented transient dysphagia, which recovered after a few months rehabilitation period. Neurological symptoms relieved significantly in the long-term follow-up in comparison to preoperative status; however, dizziness episodes still appear occasionally. The subependymoma is a low-grade glioma, with a well-known good survival prognosis, and low chances of recurrence when complete resection is accomplished; therefore, the patient did not required additional therapy after surgery (Figures 1A,B).

#### Case 2

A 30-year-old man had a 2-month history of headaches, vomiting, gait ataxia, dysphonia, dysphagia, and urinary incontinence presenting a 60 KPS. T1-weighted cerebral MRI scans showed a wider medulla due to a hypointense heterogeneous mass with necrosis or polimicrocysts. Gadolinium-enhanced T1-weighted MRI scans demonstrated a heterogeneously enhancing mass. MRI scans of the complete neuroaxis were unremarkable. The patient had had a suprasellar germ cell tumor in childhood that was treated with radiotherapy. He did well in the long-term follow-up, without evidence of recurrence during the last 20 years. Blood markers as alfa-fetoprotein (AFP), β-human chorionic growth factor (BHCG), and embryonic-carcinoid antigen (ECA) levels were within the normal range. Lumbar puncture to obtain CSF biochemical markers (AFP, BHCG, and ECA) or pathological cells was inadvisable because of the tumor extension and the presence of intracranial hypertension.

**Figure 2:**
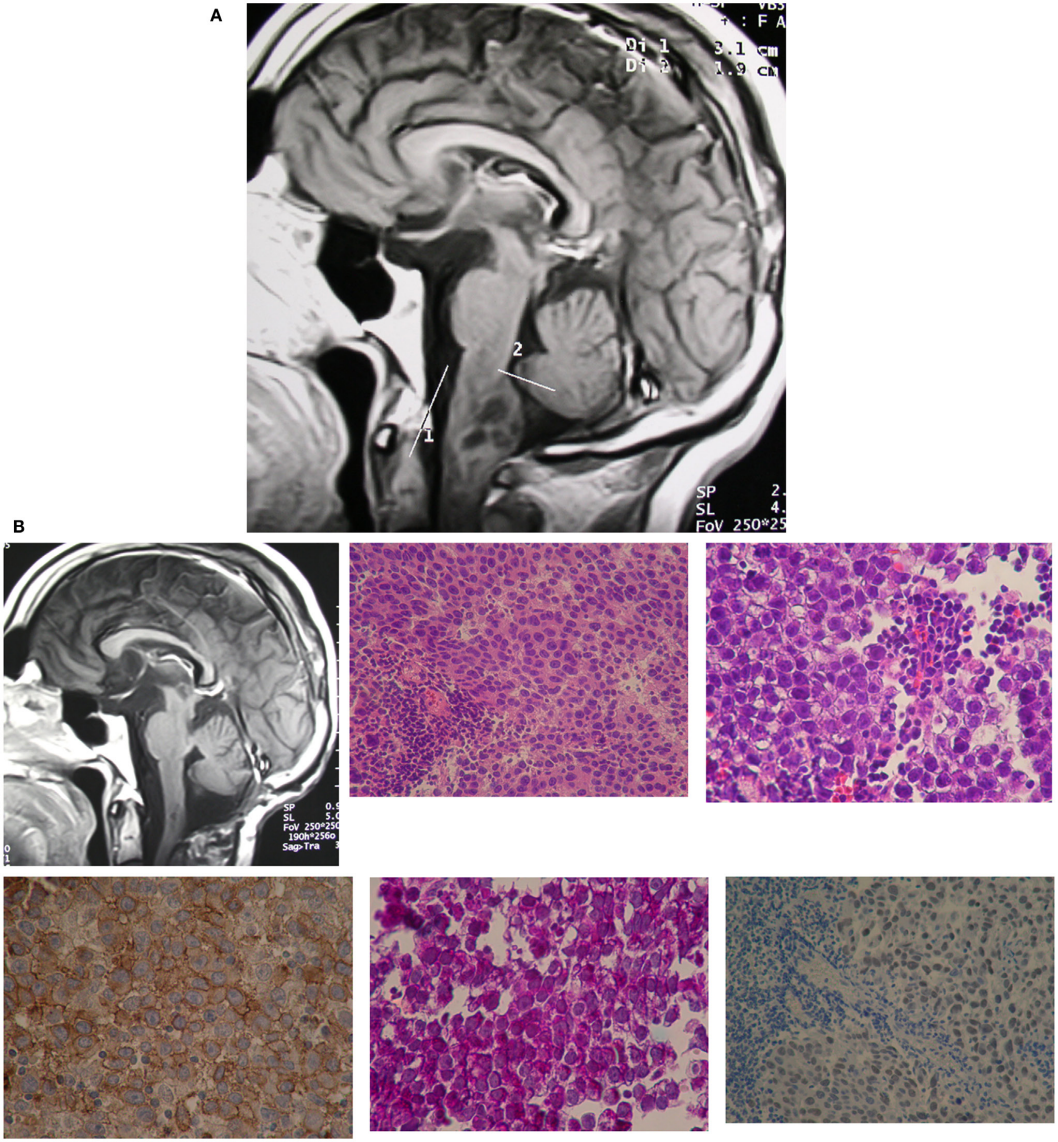
**(A)** Case 2. Preoperative T1-weighted MRI scans showed a wider MO due to a hypointense heterogeneous mass with necrosis or polimicrocystic component. Gadolinium-enhanced T1-weighted MRI scans demonstrated a heterogeneous enhancing mass. **(B)** T1-weighted brain MRI scans showed postoperative result.

A direct approach was performed with the presumptive diagnosis of a new CNS lesion or a progression of the previous disease. The intraoperative finding demonstrated a germ cell tumor and, as this type of tumor is very sensitive to oncological treatment, only a subtotal resection was performed, No surgical complications were observed. The neurological status dramatically improved postoperatively to 90 KPS; it could probably be related to the evacuation of the cyst component of the tumor and the posterior fossa decompression. Then, he received chemotherapy and radiation therapy (Figures 2A,B).

### Surgical Technique

**Figure 3:**
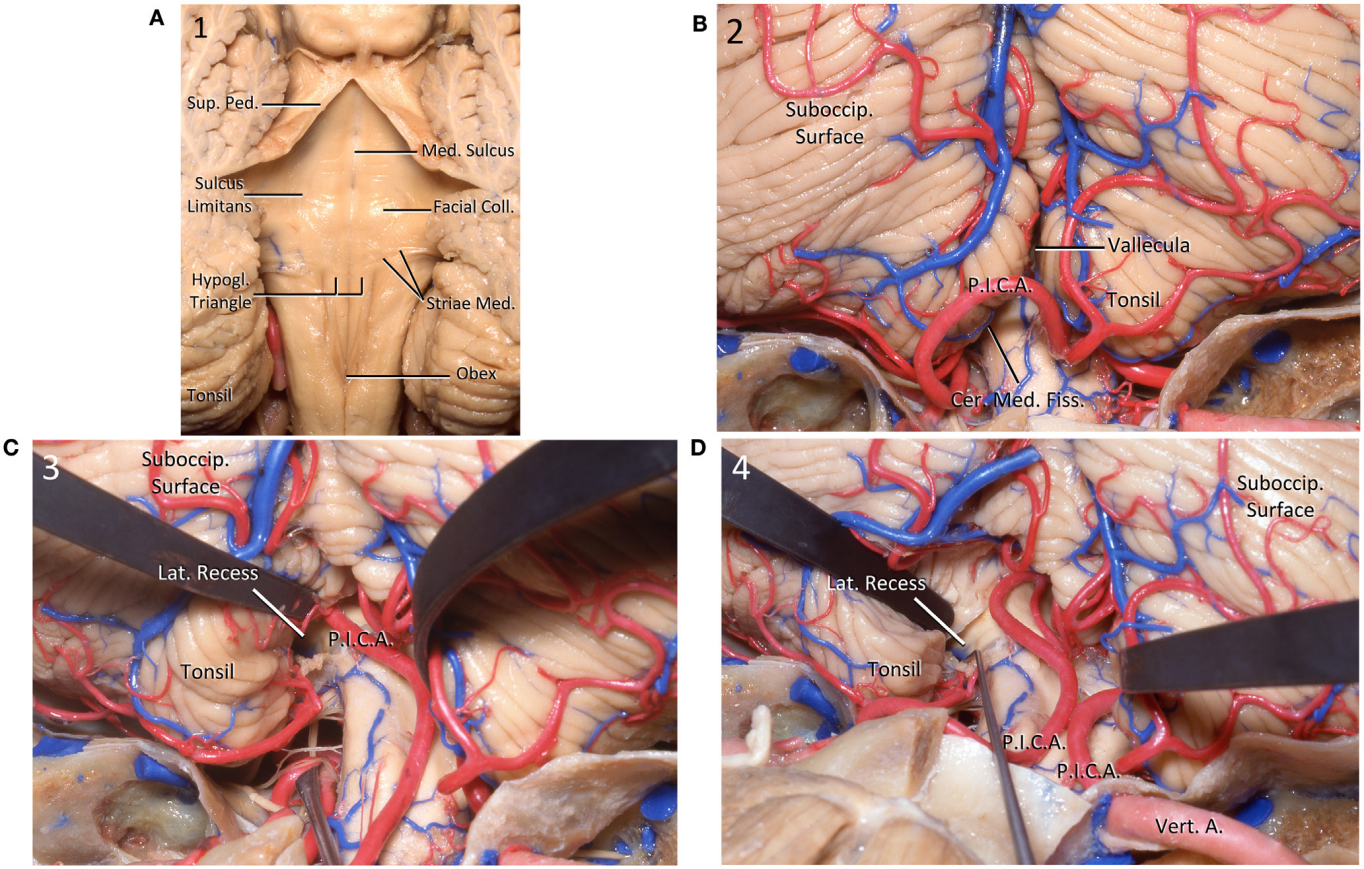
**(A)** Posterior view of the floor of the IV ventricle. The floor is divided in the midline by the median sulcus and craniocaudally into pontine, junctional, and medullary parts. *Coll*., colliculus; *Hypogl*., hypoglosal triangle; *Med*., median, medullary; *Ped*., peduncle; *Sup*., superior. **(B)** Posterior view of the cerebellum. *Cer*., cerebello; *Fiss*., fissure; *Med*., medullary; *P.I.C.A*., posteroinferior cerebellar artery; *Suboccip*., suboccipital. **(C)** Posterior view of the cerebellum. The left tonsil has been retracted superolaterally to expose the surface of the medulla as well as the lateral recess of the IV ventricle. *Lat*., lateral; *P.I.C.A*., posteroinferior cerebellar artery; *Suboccip*., suboccipital. **(D)** Posterior view of the cerebellum. A dissector is located into the lateral recess. *A*., artery; *Lat*., lateral; *P.I.C.A*., posteroinferior cerebellar artery; *Suboccip*., suboccipital; *Vert*., vertebral.

We describe the surgical technique, supporting description of approach with some anatomical specimens performed in laboratory (Figures 3A–D).

The patient is positioned prone with the head placed in a neutral position whereas the cervical spine is slightly anteriorly flexed. Bilateral somatosensory evoked potential and VIII, XI and XII cranial nerves monitoring are performed. A midline vertical skin incision is made from the inion to the second vertebra. A suboccipital midline craniotomy extended to the posterior rim of the foramen magnum is performed. The posterior arch of C1 is removed, but preserving the atlantooccipital joints.

**Figure 4:**
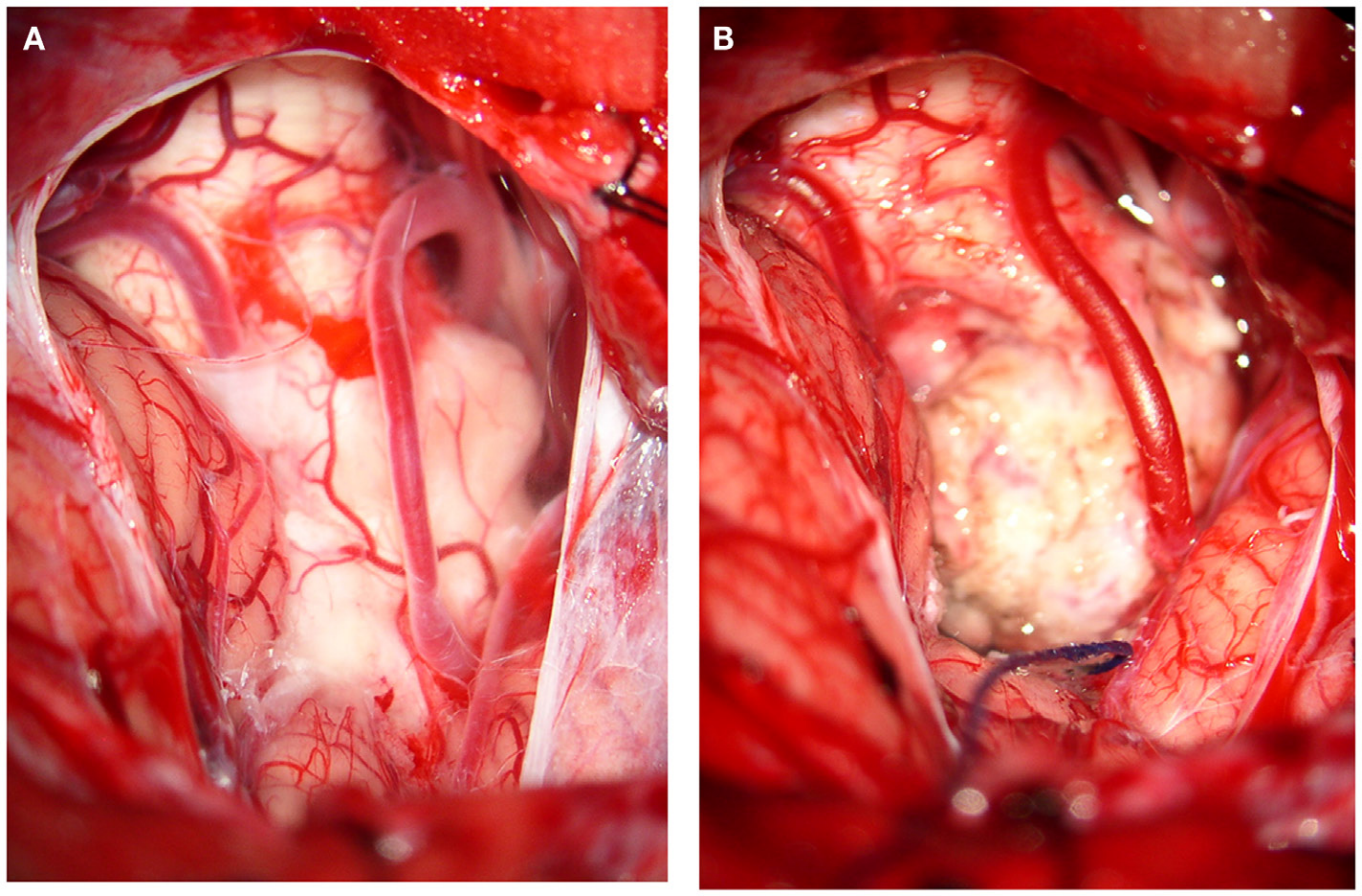
**Illustrative images of surgery**. Case 1: MO subependymoma. **(A)** The subtonsillar approach showed the cerebellar vermis and the cerebellar tonsils; dense arachnoidal adherences covered partially the PICAs and also the intrinsic and in part the exophytic MO tumor. **(B)** After a sharp dissection of arachnoidal adherences, the tumor was completely removed with preservation of both posterior inferior cerebellar arteries (PICAs).

The dura is opened in Y-like fashion. Cerebellomedullary arachnoid is sharply and meticulous dissected unilaterally to allow mobilization of cerebellum’s tonsils off the dorsal surface of the medulla. Opening of the tonsillovermian fissure further increased the exposure. This maneuver allows the access to the inferior end of the IV ventricle and the MO. The medulla enlarged by the intrinsic lesion, is displacing the tonsils upwards. The bulging neural lesion is displacing the posterior inferior cerebellar arteries (PICAs), and the vascular structures are identified and preserved. Regarding veins, the cerebellomedullary fissure vein drains the superior and ventral surfaces of the tonsils and portions of the inferior vermis. It passes laterally within the fissure to empty into the tributaries of the superior petrosal sinus. At its medial origin, the cerebellomedullary fissure veins may communicate by means of the lateral uvular vein with the inferior vermian vein. The posterior aspect of the medulla can be exposed without sacrificing neural tissue. A minimal neural opening is performed in the midline, in the area of less tissular vascularization, known as safe entry zone of the inferior half of the medulla. Removal of small piece of tissue for histopathological study is performed in real time to define the extent of resection. Then, the duramater is closed in watertight fashion. Muscles and superficial tissues are closed in the usual manner (Figures 4A,B).

## Results

Two patients with masses located in the inferior half of MO are reported. One was a focal lesion and the other was an exophytic one. The suboocipital subtonsillar midline approach used in these two cases provided a wide surgical view to the foramen of Luschka laterally and up to the middle cerebellar peduncle. It was found that the tumors had produced a mild elevation of the tonsils making the access easier, and there was no need to use retractors. The approach provided enough decompression and simultaneously allowed to obtain histological samples. The extent of resection depended to the intraoperative histopathological findings. Good neurological results were achieved without complications related to the procedure.

## Discussion

Lesions located in the MO represent undoubtedly a difficult challenge, since the expected high risk of surgical exploration of this eloquent area. Moreover, their low incidence in adults combined with little worldwide experience has provided scarce information about surgical results and prognosis. This has probably discouraged the treatment of tumors in this localization for many years.

Although modern neurological imaging techniques provide well-defined images, the histological diagnosis cannot be certainly confirmed. It has been reported that neurorradiological assessment varied from histopathological diagnosis in approximately 40% of brain stem cases (21–23). Despite the high rate of agreement found between MR spectroscopy and histopathology, there might be a high rate of bias in this location, especially for the partial volume effect of adjacent normal brain tissue and/or cerebrospinal fluid (8, 24, 25). Therefore, neuroimaging techniques are not so reliable as histological diagnosis, particularly in this area of the brain.

Although stereotactic biopsy is usually safely implemented in other areas of BS such midbrain or pons, pure MO lesions are seldom biopsied (26). For instance, one of the largest last decade series reported 20 stereotactic biopsies of brainstem tumors in children but none for MO (27). We believe that lacks sufficient experience in the literature about stereotactic biopsy in MO and consequently, it is recommended to discuss case by case. Direct surgery could avoid the potential risks of stereotactic biopsy in this particular area of the CNS.

Regarding the subtonsillary approach, it was originally described to reach the fourth ventricle, and anatomical details have previously reported (11–15, 28, 29). The access to lesions located in the inferior half of the medulla is particularly wide and safe, with the assistance of intraoperative neurophysiological monitoring. Surgical planning using diffusion tensor imaging and tractography, although not used in the cases reported here, will surely contribute to develop surgical strategies in the near future as evidenced in other eloquent areas of CNS (30, 31). The advantages of the dissection of the tonsils over the transvermian approach is that the dissection of the cerebellomedullary arachnoid and the tonsillovermian fissure, allow the preservation of the venous structures specially the cerebellomedullary fissure veins that drains the superior and ventral surfaces of the tonsils and a portion of the inferior vermis. Also, the exposure of the posterior aspect of the MO does not require the opening of additional neural tissue of the inferior vermis, providing enough room to reach to a safe entry zone in the inferior half of the medulla. It is also likely that the extensive arachnoidal dissection decreases the risks of hydrocephalus. Other approach, such as the far lateral suboocipital, should only be considered for lesions located in the anterolateral aspect of MO.

The broader variety of pathological findings that could be found in the MO supports the benefit of getting an accurate histological diagnosis. Regarding the role of intraoperative pathology reviewed in real time, although it can not always confirm the definitive histopathological diagnosis in the operating room, usually provides enough information to determine the extent of resection.

In conclusion, direct surgery via subtonsillar approach assisted by intraoperative neurophysiological monitoring provides a safe surgical way to this critical area of the brain. The awareness of the histopathology is decisive to define the course of treatment and could avoid the disastrous consequences of empiric treatments in cases appropriately selected.

## Ethics Statement

All subjects gave written informed consent according to the requirements of the Institute of Medical Research A. Lanari, University of Buenos Aires.

## Author Contributions

AR: design of the research, writing of the article, revision of the literature. DH: participation in the design of the research and critical review of the paper. AC: work in the Anatomical Research Laboratory.

## Conflict of Interest Statement

The authors declare that the research was conducted in the absence of any commercial or financial relationships that could be construed as a potential conflict of interest.

## Abbreviations

BS, brainstem; CNS, central nervous system; CSF, cerebrospinal fluid; MO, medulla oblongata; MRI, magnetic resonance imaging; PICA, posterior inferior cerebellar artery.
